# Hydrophobic amino acids as a new class of kinetic inhibitors for gas hydrate formation

**DOI:** 10.1038/srep02428

**Published:** 2013-08-13

**Authors:** Jeong-Hoon Sa, Gye-Hoon Kwak, Bo Ram Lee, Da-Hye Park, Kunwoo Han, Kun-Hong Lee

**Affiliations:** 1 Department of Chemical Engineering, Pohang University of Science & Technology, San 31, Hyoja-Dong, Nam-Gu, Pohang-Si, Gyeongbuk 790-784, Korea; 2 Center for Hydrate Research, Chemical and Biological Engineering Department, Colorado School of Mines, Golden, CO 80401, USA; 3 Energy Laboratory, Samsung Advanced Institute of Technology, San 14, Nongseo-Dong, Giheung-Gu, Yongin-Si, Gyeonggi-Do 446-712, Korea; 4 CO_2_ Project Team, Research Institute of Industrial Science & Technology, San 32, Hyoja-Dong, Nam-Gu, Pohang-Si, Gyeongbuk 790-600, Korea

## Abstract

As the foundation of energy industry moves towards gas, flow assurance technology preventing pipelines from hydrate blockages becomes increasingly significant. However, the principle of hydrate inhibition is still poorly understood. Here, we examined natural hydrophobic amino acids as novel kinetic hydrate inhibitors (KHIs), and investigated hydrate inhibition phenomena by using them as a model system. Amino acids with lower hydrophobicity were found to be better KHIs to delay nucleation and retard growth, working by disrupting the water hydrogen bond network, while those with higher hydrophobicity strengthened the local water structure. It was found that perturbation of the water structure around KHIs plays a critical role in hydrate inhibition. This suggestion of a new class of KHIs will aid development of KHIs with enhanced biodegradability, and the present findings will accelerate the improved control of hydrate formation for natural gas exploitation and the utilization of hydrates as next-generation gas capture media.

As the energy crisis and increasing levels of environmental pollution are being addressed as the major challenges affecting the modern world, mankind has been steadily seeking new alternative clean energy resources^1^, including hydrogen^2^,^3^,^4^, solar^5^, geothermal^6^, wind^7^, and biomass energies^8^. However, there is still no solution that adequately meets the rapidly increasing energy demands of the world. Instead, shale gas reached recent headlines as it became commercially available from technological advances in horizontal drilling and hydraulic fracturing. It has been estimated that shale gas could supply decades of use for worldwide energy consumption^9^. In a similar context, gas hydrates, crystalline water-based solids in which gas molecules are enclathrated in a framework linked by hydrogen bonded water molecules^10^, as promising energy resources are within reach^11^,^12^. The foundation of the energy industry now moves towards gas.

Accordingly, the pipeline transportation technology is becoming increasingly significant due to a vast amount of the gas fuel production and CO_2_ emission from the gas fuel combustion. One important consideration is that gas and oil transportation lines often provide favorable temperature and pressure conditions for gas hydrate formation, resulting in a build-up of hydrates and subsequent pipeline blockage^13^. This results in significant financial losses for gas and oil companies due to the necessary pipeline shutdown and recovery, in addition to the potential for huge explosions such as those that were responsible for the Piper Alpha oil rig disaster (1988) and the Gulf of Mexico oil spill (2010). Safety and environmental issues derived from such accidents are clearly of major concern throughout the world. Flow assurance for natural gas transportation and CO_2_ sequestration becomes one of the most challenging area in the world energy industry^14^.

One promising technology to overcome this problem involves the injection of hydrate inhibitors into the pipelines. Traditionally, thermodynamic hydrate inhibitors (THIs) such as alcohols have been used to shift the formation conditions to lower temperature and higher pressure regions^10^. However, due to economic and environmental concerns, kinetic hydrate inhibitors (KHIs) are currently receiving a great deal of attention as alternatives^15^,^16^. KHIs delay nucleation and/or retard growth of hydrates at low dose (less than 1 wt%). Conventional KHIs include polymers^17^,^18^, antifreeze proteins^19^,^20^, ionic liquids^21^, and quaternary ammonium zwitterions^22^. However, despite decades of research, investigations into the principles of gas hydrate inhibition were hardly achieved despite their industrial and academic significance^14^,^15^,^16^,^23^. Also, there is still a need for development of environmentally friendly KHIs with enhanced biodegradability due to the potential environmental risks. The major key for flow assurance lies within a fundamental understanding of the mechanisms involved in hydrate formation and inhibition by KHIs^24^.

In the past, the mechanism of hydrate inhibition was interpreted in terms of an adsorption inhibition hypothesis (Fig. 1b), with adsorption of KHIs on the hydrate surface being a key process in the inhibition^25^. It was hypothesized that polyvinyl pyrrolidone (PVP), a well-known KHI, inhibits hydrate formation by adsorption through hydrogen bonds^26^,^27^. Accordingly, KHI abilities of a variety of polymers^18^,^28^,^29^,^30^,^31^,^32^,^33^,^34^,^35^,^36^ on hydrate inhibition and morphological changes^17^,^37^ of hydrate crystals induced by the adsorption of KHIs have been reported. However, a more recent study demonstrated that PVP has no direct contact with the hydrate surface^38^,^39^, raising the possibility that adsorption is not the only mechanism of hydrate inhibition. Recently, a two-step mechanism was proposed, involving perturbation of the local water structure (Fig. 1c), thus increasing the barrier to nucleation^40^. In contrast to the case of the adsorption inhibition hypothesis, there are no reports on experimental investigations into the perturbation inhibition hypothesis, only a few simulation studies^22^,^41^.

In the present work, we propose natural hydrophobic amino acids, which are naturally occurring molecules, as novel KHIs. Owing to amphiphilic nature, charged molecular structure, and inherent eco-friendliness, such molecules have great promise for this application. In addition, amino acids with hydrophobic side chains can serve as a model system for investigating hydrate inhibition phenomena, as a variety of physicochemical properties can be achieved by systematically selecting the appropriate molecule. Thus, it was hypothesized that fundamental insights into hydrate inhibition could be gained by correlating the effects of amino acids on heterogeneous nucleation and growth with their physicochemical properties. Here, we report CO_2_ hydrate formation kinetics for heterogeneous nucleation at the onset (stage 1) of hydrate formation, and growth during (stage 2) and after (stage 3) hydrate formation.

## Results

### Hydrophobic amino acids

Amino acids are one of the major building blocks that make up life on earth. There are 20 distinct naturally occurring molecules, each containing a carboxylic acid, an amine, and a unique side chain group, which combine to make up the proteins found in living organisms. Owing to the nature of their origin, they are environmentally friendly and biodegradable. Their physical and chemical properties are strongly dependent on the particular side chain, which can vary from a simple apolar alkyl chain (hydrophobic) to a positively or negatively charged moiety (hydrophilic).

There are two interesting features of hydrophobic amino acids that contribute to their potential to be used as KHIs. First, from the calculations, the majority of amino acids were found to behave as zwitterions under CO_2_ hydrate forming conditions (Table 1). The electric charge on these molecules enables them to interact with water molecules through strong electrostatic interactions. In addition, it has been found that water molecules around electric charges become less “icelike”^42^. The second feature is that while the amino acids used in this work are termed hydrophobic, they also have hydrophilic character due to their carboxylic acid and amine groups. Hence, they can interact with water molecules through strong hydrogen bonds.

An important consideration when investigating hydrophobic amino acids as KHIs is their perturbation of the local water structure. It was demonstrated from direct experimental evidence using infrared spectroscopy^43^, neutron scattering^44^, and Raman scattering^45^ that the hydrogen bond network between water molecules around hydrophilic moieties of hydrophobic amino acids was disrupted, while that around hydrophobic alkyl chains was strengthened. The extent of these perturbations are correlated well with the hydrophobicity of the amino acids^43^,^45^. Interestingly, a similar result has been reported for quaternary ammonium zwitterions, with similar physicochemical properties to hydrophobic amino acids^42^. A different local water structures around the hydrophilic and hydrophobic ends were reported. Along with this, hydrophobic amino acids have great potential as a model system for investigating the gas hydrate inhibition mechanism owing to the ease with which the balance between their hydrophilicity and hydrophobicity can be easily controlled, thus varying the degree of perturbation of the local water structure around amino acid molecules.

### Stage 1. Heterogeneous nucleation kinetics

Nucleation kinetics measurements were used to observe the onset of hydrate formation. Hydrate nucleation depends on the displacement from equilibrium (subcooling temperature), state/history of the water, impurities, composition of the gas, degree of agitation or turbulence, and geometry of the system or surface area^10^. Thus, in laboratory evaluations, these factors need to be controlled with great precision. In general, the induction time is a direct indicator of how efficient KHIs are in delaying hydrate nucleation under possible hydrate forming conditions. However, the induction time may range from seconds to days or longer due to the stochastic nature of hydrate nucleation^46^. As expected, induction time measurements (see Supplementary Figs. S2a and S2b) yielded results that were too variable to enable differentiation of inhibition performances. As a more accurate alternative, subcooling temperatures at the onset of hydrate nucleation were measured in order to evaluate the KHIs. Previous studies have established that measurements of subcooling temperatures are less variable than those of induction times^10^,^47^.

**Figure 2:**
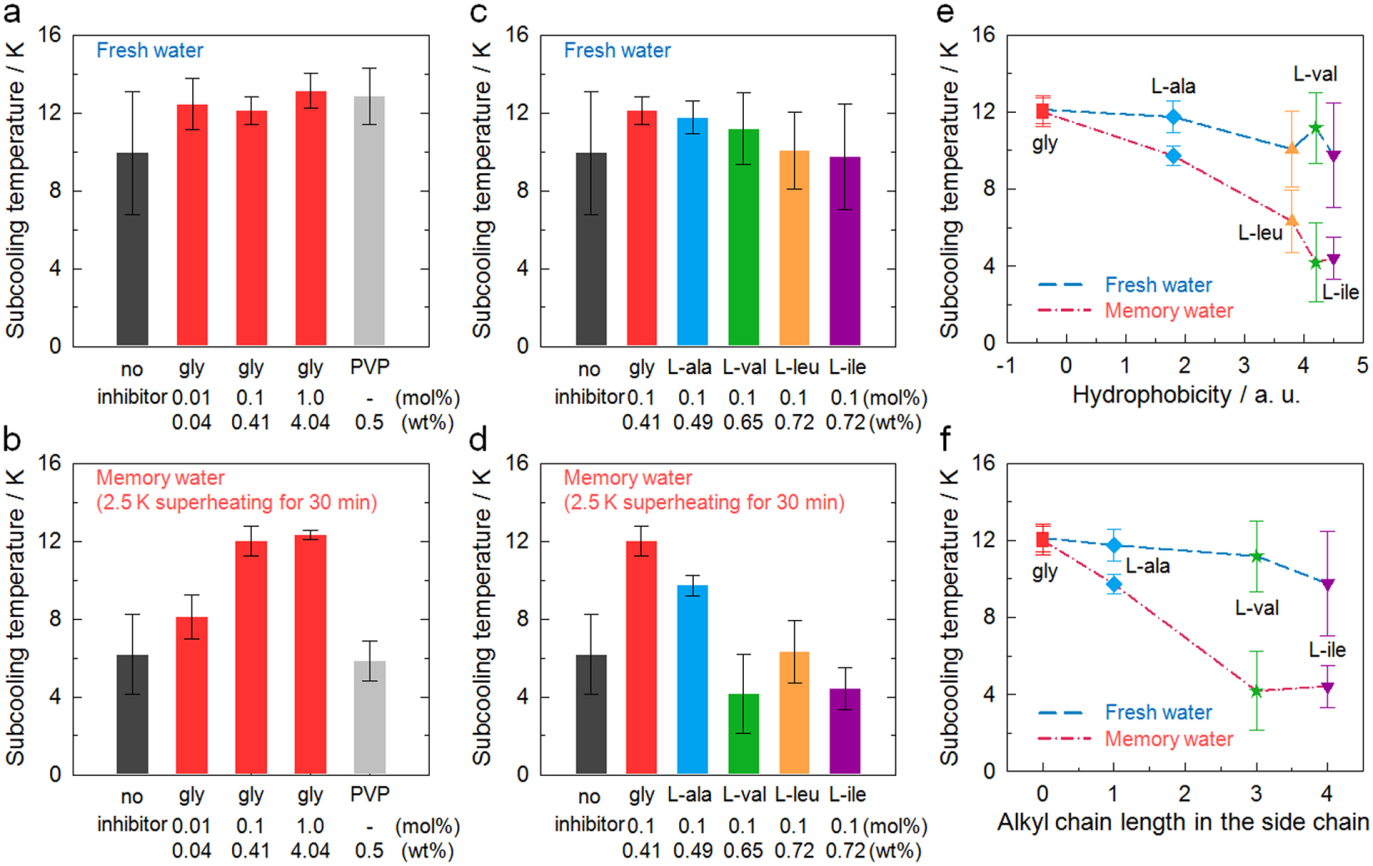
Heterogeneous nucleation kinetics of the CO_2_ hydrates in the presence of hydrophobic amino acids. Subcooling temperatures at the onset of CO_2_ hydrate nucleation with glycine or PVP in (a) fresh water and (b) memory water, and with hydrophobic amino acids in (c) fresh water and (d) memory water. Memory water was prepared by dissociating preformed CO_2_ hydrate at a temperature 2.5 K above the equilibrium temperature for 30 min. Measured subcooling temperature were correlated with (e) hydrophobicity and (f) alkyl side chain length for the hydrophobic amino acids. The values of the average and standard deviation are shown.

On the addition of 0.01 mol% glycine to the system, the average subcooling temperature increased by more than 2 K (Fig. 2a), which is a notable difference since the probability of hydrate nucleation exponentially increases as the temperature is lowered at high subcooling. On increasing the glycine concentration from 0.01 to 1.0 mol%, the average subcooling temperature remained almost constant. These results demonstrate that, in fresh water, glycine has the potential to be used as a KHI, with performance comparable to PVP. However, a different trend was observed when the same measurements were carried out in memory water due to the “memory effect”. The memory effect is a phenomenon where hydrates form more easily from gas and water with previous hydrate history^10^. This phenomenon is attributed to either the presence of residual hydrate structure including partial hydrate cages and polyhedral clusters or the dissolved gas molecules remained after hydrate dissociation^10^. The average subcooling temperature in the absence of the KHI was lower than that in fresh water, and the measured temperatures were less variable, which is consistent with previous reports^48^. Measuring subcooling temperatures in memory water enabled a more reproducible and reliable evaluation of KHIs to be obtained^48^. Interestingly, as the concentration of glycine was increased from 0.01 to 1.0 mol%, the average subcooling temperature also increased, while PVP did not delay hydrate nucleation to any extent. This result indicates that glycine has the ability to eliminate the memory effect, which agrees with a similar result previously reported for antifreeze proteins^19^,^20^.

The nucleation kinetics in the presence of different hydrophobic amino acids were investigated (Figs. 2c and 2d). As the length of the alkyl side chain of the amino acids increased, thus enhancing hydrophobicity, the inhibition performance decreased. For the most hydrophobic of the molecules tested, L-leucine and L-isoleucine, there was no significant effect on hydrate inhibition. This result indicates that longer alkyl chains adversely affect the performance of hydrophobic amino acids as KHIs. Interestingly, this tendency is completely opposite to that observed when using hydrophobic amino acids as THIs^49^. In this case, hydrophobic amino acids with longer alkyl side chains were better THIs due to the enhanced hydrophobic effect^49^. The differences between the KHI abilities of amino acids were more remarkable in the measurements carried out in memory water (Fig. 2d). While L-alanine was found to be slightly less efficient than glycine, amino acids with longer alkyl chains performed poorly, with some of them even observed to accelerate hydrate nucleation. Therefore, it appears that there is a critical alkyl chain length that determines the kinetic inhibition performance of the hydrophobic amino acids, with those with shorter alkyl chains acting as better KHIs. The nucleation inhibition performances of the molecules were correlated well with their hydrophobicity and alkyl side chain length, especially in the experiments carried out in memory water (Figs. 2e and 2f).

### Stage 2. Growth kinetics

Measurements of growth were employed to assess the intermediate stage of hydrate formation. Although mass and heat transfer are critical factors in determining the rate of hydrate growth^10^, the factors that affect hydrate nucleation should also be considered. Here, the hydrate growth rate was directly determined by measuring gas uptake.

**Figure 3:**
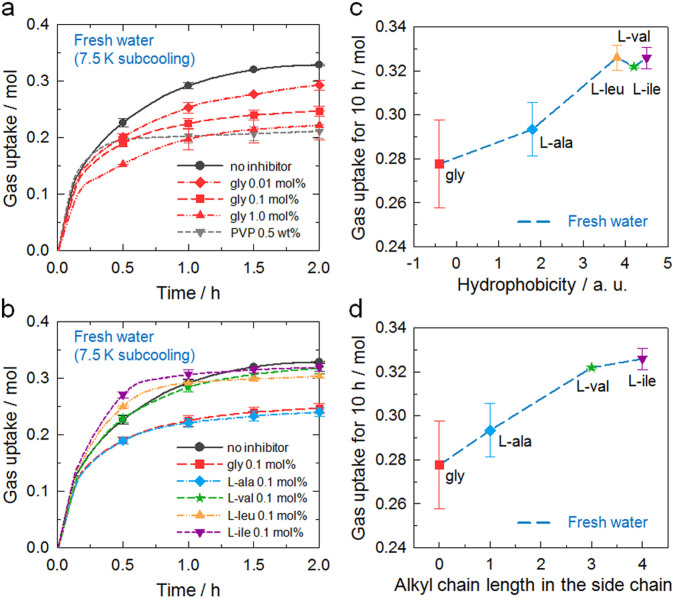
Growth kinetics of the CO_2_ hydrates in the presence of hydrophobic amino acids. Gas uptake rates during CO_2_ hydrate growth (a) with glycine or PVP and (b) with hydrophobic amino acids. Measurement of hydrate growth was initiated just after the onset of hydrate nucleation. The average values of gas uptake were calculated from several repeating measurements taken every 10 s. The symbols and their error bars indicate the average and standard deviations of gas uptake at each time point, respectively. Measured gas uptake rates were correlated with (c) hydrophobicity and (d) alkyl side chain length for the hydrophobic amino acids.

In the system without any KHI (Fig. 3a), extremely rapid growth immediately followed the onset of hydrate nucleation, with the growth rate then decreasing steadily over time. The gas uptake curve became almost saturated approximately 2 h after the initiation of hydrate growth. On increasing the glycine concentration from 0.01 to 1.0 mol%, the growth rate decreased further. While PVP did not retard the hydrate growth during the first 15 min, the gas uptake rate underwent a significant decrease after this point and there was very little further increase in gas uptake up to 10 h (see Supplementary Fig. S3a). Taking previously reported hydration values of CO_2_ hydrates^50^,^51^, calculated conversion ratio of hydrate to water after hydrate growth finished ranged from 61.3% to 76.0%. The performance of glycine as a KHI on hydrate growth was comparable to PVP overall, although they displayed different behaviors in hydrate growth inhibition.

The measurements were repeated with the addition of 0.1 mol% of the different hydrophobic amino acids (Fig. 3b). L-alanine considerably reduced the growth rate, with a performance highly similar to glycine. However, as the length of the alkyl chain was increased further, the level of inhibition decreased. L-leucine and L-isoleucine were even observed to accelerate the hydrate growth in the early stages. It is clear that above a critical chain length, hydrate growth inhibition was adversely affected, with this trend being similar to that observed in the nucleation measurements. The growth inhibition performances of the amino acids also correlated well with hydrophobicity and alkyl side chain length (Figs. 3c and 3d).

### Stage 3. Synchrotron powder X-ray diffraction (PXRD)

PXRD analysis enabled phase identification after the formation of the hydrate. XRD is a promising technique for identifying the crystal structure of CO_2_ hydrates and their lattice parameters. It is also possible to differentiate a hydrate phase from an ice phase, making it feasible to investigate the inhibition performances of KHIs by comparing the relative amounts of ice phase to hydrate phase involved.

**Figure 4:**
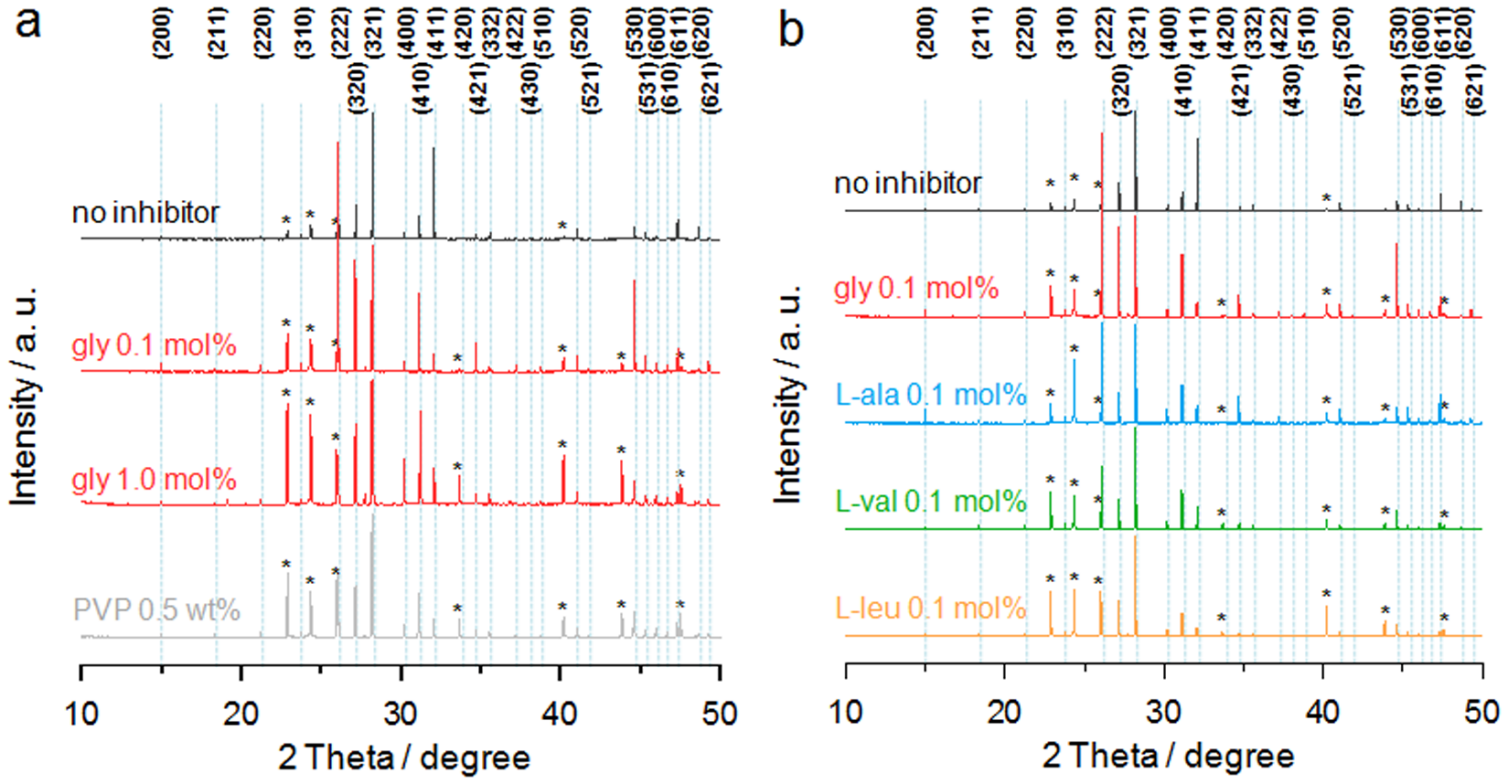
PXRD patterns of the CO_2_ hydrates in the presence of hydrophobic amino acids. Synchrotron PXRD patterns for CO_2_ hydrate (a) with glycine or PVP and (b) with hydrophobic amino acids. Diffraction peaks for cubic structure I hydrate are denoted by their Miller indices, and asterisks indicate the peak positions for the ice phase. All diffraction peak intensities were therefore normalized to the (321) peak in order to enable a comparison of their relative intensities.

The pure CO_2_ hydrate formed a cubic structure I with a *Pm3n* space group, as expected^10^, and the (321) peak had the maximum intensity (Fig. 4a). Addition of KHIs did not alter the crystal structure of the CO_2_ hydrate. The calculated lattice parameters for CO_2_ hydrate with KHIs are summarized in Supplementary Table S3, and are seen to be consistent with previously reported values^51^,^52^. Interestingly, in the presence of the glycine, several additional diffraction peaks for the ice phase were observed, which was attributed to water molecules remaining in the liquid phase freezing rather than forming hydrate. On increasing the glycine concentration from 0.01 to 1.0 mol%, the relative peak intensities for the ice phase compared to the hydrate phase became much higher, with this dependence on concentration being similar to that observed in laboratory evaluations. It is evident that the conversion of water or ice to hydrate was retarded by glycine, which is in agreement with previous reports on other KHIs^53^,^54^. On the addition of 0.5 wt% PVP, a similar diffraction pattern was obtained, and the intensities of the ice phase peaks were found to be intermediate between the 0.1 mol% and 1.0 mol% glycine samples.

In Fig. 4b, it can be seen that the CO_2_ hydrate formed in the presence of L-alanine exhibited a similar diffraction pattern to that of the glycine sample. However, contrary to expectations, the samples with L-valine or L-leucine showed a similar trend in relative peak intensities even though these amino acids were identified not to be efficient KHIs in the laboratory evaluations. This result indicates that all hydrophobic amino acids were effective in inhibiting the conversion of water or ice to hydrate, independent of their hydrophobicity, after a long period of hydrate formation.

## Discussion

From measurements of nucleation kinetics (stage 1), glycine and L-alanine were identified as being more efficient KHIs than the other amino acids tested, and an ability to eliminate the memory effect was also observed. The only difference between the individual hydrophobic amino acids is the level of hydrophobicity due to their alkyl chains. Their ionization characteristics are almost identical as they do not contain any other charged groups other than the terminal carboxylic acid and amine moieties. The hydrogen bond network surrounding these terminal groups is expected to be disrupted, with the local water structure being incompatible with the structure of the hydrate surface^22^,^43^,^44^,^45^. However, as the length of the alkyl chain increases, thus enhancing hydrophobicity, the strengthening effect on the local water structure becomes increasingly significant. Thus, based on the correlation between the hydrophobicity of amino acids and their inhibition performances (Figs. 2e and 2f), it is quite plausible that perturbation of the local water structure plays an important role in inhibiting heterogeneous nucleation.

**Figure 5:**
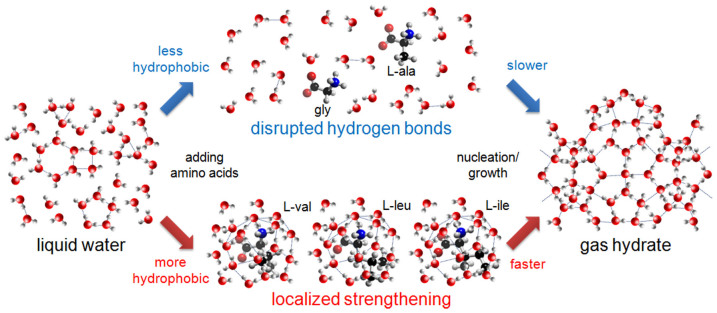
The representation of the different hydrate inhibition processes driven by hydrophobic amino acids. Less hydrophobic amino acids (glycine and L-alanine) disrupt hydrogen bonds between water molecules to inhibit hydrate formation while more hydrophobic amino acids (L-valine, L-leucine, and L-isoleucine) strengthen the local organization of the water structure.

In the growth kinetics measurements (stage 2), two fundamental insights into the mechanism of hydrate inhibition were gained. Firstly, the relationship between amino acid hydrophobicity and nucleation inhibition followed the same trend as for growth inhibition (Figs. 3c and 3d), while there have been several recent reports on differences in effectiveness of KHIs on nucleation and growth^55^,^56^. This result is remarkable in that nucleation inhibitors interact with the foreign nucleating solid impurities, whereas growth inhibitors interact with the hydrate surface^55^. However, perturbation characteristics of the water structure are independent of the surface properties. Thus, it is likely that perturbation of the water structure plays a more important role than adsorption in hydrate inhibition when using hydrophobic amino acids as KHIs. Secondly, an important consideration is the distinct shape of the gas uptake curves. For the systems with no inhibitor or PVP, the rate of hydrate growth gradually decreased over time (Fig. 3a). In the glycine system, however, an inflection point was observed approximately 7 min after hydrate growth started, and then the slope of the gas uptake curve suddenly decreased. In addition, L-leucine and L-isoleucine in particular showed additional inflection points in the growth curve at around 35 min (Fig. 3b). The difference in the inhibitory behavior on growth kinetics provides additional experimental evidence in support of the hypothesis that amino acids may have a growth inhibition mechanism different from that of PVP which is mainly driven by adsorption. Therefore, it is suggested that the balance between the effects of the hydrophilic terminal groups and the hydrophobic side chains on the local water structure determines their effectiveness in terms of growth inhibition, as well as nucleation inhibition, with the key process being perturbation of the local water structure (Fig. 5).

From the synchrotron PXRD analysis (stage 3), the relative peak intensities for the ice phase compared to the hydrate phase of the CO_2_ hydrates in the presence of the KHIs were much higher than for the system without inhibitor, and the concentration dependence was similar to that observed in laboratory evaluations. However, all hydrophobic amino acids tested were found to be efficient in retarding the conversion of water or ice to hydrate after a long period of hydrate formation, and the influence of hydrophobicity on the inhibition performances was inconsistent with that found in laboratory evaluations. Further investigations on hydrate dissociation kinetics in the presence of amino acids will be beneficial for achieving greater understanding of hydrate inhibition phenomena.

In conclusion, we have proposed natural hydrophobic amino acids as a new class of KHIs, and confirmed their abilities to inhibit gas hydrate formation. By correlating hydrophobicity with the inhibition performance of amino acids, we found that perturbation of the water hydrogen bond network by KHIs plays a critical role in hydrate inhibition. Therefore, it would be beneficial for future work on the investigation of the hydrate inhibition mechanism to consider the perturbation and adsorption phenomena as complementary to one another. The environmentally friendly nature of amino acids means that they pose negligible contamination risk to the surrounding area, and the ease with which they can be mass-produced makes them more cost-effective than conventional KHIs. This new class of KHIs, and the identification of the fundamental mechanisms by which they work should aid the further development of KHIs with enhanced biodegradability. The present findings will accelerate the achievement of improved control of hydrate formation and dissociation for natural gas exploitation, and the utilization of hydrates as next generation gas storage materials and transportation media.

## Methods

### Macroscopic measurements

The cell (see Supplementary Fig. S4) was charged with 60 g water with or without KHIs. After immersing the cell in an ethanol bath, it was flushed with CO_2_ and pressurized up to 36 bar at 284.05 K with agitation of 450 rpm. The cell was maintained under these conditions for at least 3 h to allow an equilibrium state to be reached. Macroscopic measurements were performed using 3 different methods: isothermal, constant cooling, and constant cooling with superheated hydrate (see Supplementary Fig. S5).These methods have been frequently used to assess the performance of KHIs on a laboratory scale^47^.

For the isothermal method, the system was cooled to 273.45 K at the maximum cooling rate without agitation. The operating temperature of 273.45 K was determined to be the lowest possible for inducing rapid hydrate nucleation while remaining slightly above the freezing temperature of water in order to prevent ice formation. After the temperature was reached, agitation was applied to induce hydrate nucleation. The induction time, t_i_, is defined as: 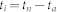
where t_a_ is the time when the agitation was initiated and t_n_ is the time when the nucleation started which was indicated by a sudden increase in temperature. After the hydrate nucleation, hydrate growth rate was measured for 10 h at constant temperature, and subsequently converted from temperature and pressure to the number of moles of gas consumed (gas uptake) by the following equation^57^: 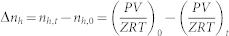
where n_h_ is the number of moles of gas consumed to form the hydrate phase or dissolved in liquid phase at time t and 0, Z is the compressibility factor calculated by Pitzer's correlation^58^, and V is the volume of vapor phase in the cell.

In the constant cooling method, the system was cooled at the maximum cooling rate with agitation. Although the system entered the hydrate forming region at approximately 281.45 K, hydrate nucleation was not initiated immediately due to the metastability. As the temperature was lowered further, hydrate nucleation was indicated by a sudden temperature increase. The subcooling temperature at hydrate nucleation in fresh water was calculated using the following equation: 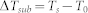
where T_0_ and T_s_ are the phase equilibrium temperatures for pure water and an aqueous solution at the onset pressure, respectively.

In the constant cooling with superheated hydrate method, the subcooling temperature at hydrate nucleation in memory water was measured. To introduce the same extent of memory to all measurements, the temperature and time applied for dissociation after hydrate formation needed to be considered^48^. First, hydrate nucleation was induced by the constant cooling method and hydrates were formed until a pressure approximately 5 bar below the onset pressure was reached. The agitation was then stopped and the temperature was quickly increased to 283.95 K, which is 2.5 K above the equilibrium temperature, over 15 min. The hydrates were dissociated without agitation until a pressure of 35 bar was reached (gas molecules corresponding to around 1 bar in the cell when completely dissociated were still enclathrated in the hydrate phase). If the same temperature and time were applied to dissociate hydrates with different additives, there may not be a similar extent of memory because of their different dissociation kinetics^59^. As the pressure reached 35 bar, the agitation was resumed and hydrates were subsequently dissociated until the pressure reached a value corresponding to complete dissociation. This step was usually complete within 10 min. The hydrates were then dissociated with agitation for an additional 30 min. These procedures enabled precise control of the extent of memory. After hydrate dissociation, hydrate nucleation was induced once again by the constant cooling method.

### Synchrotron PXRD

Using the isothermal method, hydrate was formed over 10 h until a constant pressure was reached, and then continued for 5 h more in order to induce full conversion of water and gas to the hydrate phase. The materials was then transferred to a liquid nitrogen bath and the contents were frozen for 20 min to prevent hydrate dissociation. Samples were ground into fine powders while liquid nitrogen temperature was maintained. PXRD analysis was carried out using the high-resolution powder diffraction beamline (9B) at the Pohang Accelerator Laboratory (PAL) in Korea (see Supplementary Fig. S6). The incident X-rays from synchrotron radiation were monochromatized using a silicon (111) crystal to a wave length of 1.54750 Å. A sample of powder (1.0 g) was loaded on a flat plate holder and the scan was performed at 80.0 K in step mode with a fixed time of 2 s and a step size of 0.005° from 10° to 50° with a 0.5° overlap in 2θ. Using Chekcell software, the obtained diffraction patterns were indexed and lattice parameters were calculated for each sample.

## Author Contributions

J.H.S. and B.R.L. designed experiments and performed kinetics measurements; J.H.S. and G.H.K. performed synchrotron experiments; J.H.S. and D.H.P. interpreted the results; K.H. and K.H.L. supervised the research project; and J.H.S. and K.H.L. wrote the manuscript.

## Supplementary Material

Supplementary Information Supplementary Information

## Figures and Tables

**Figure 1:**
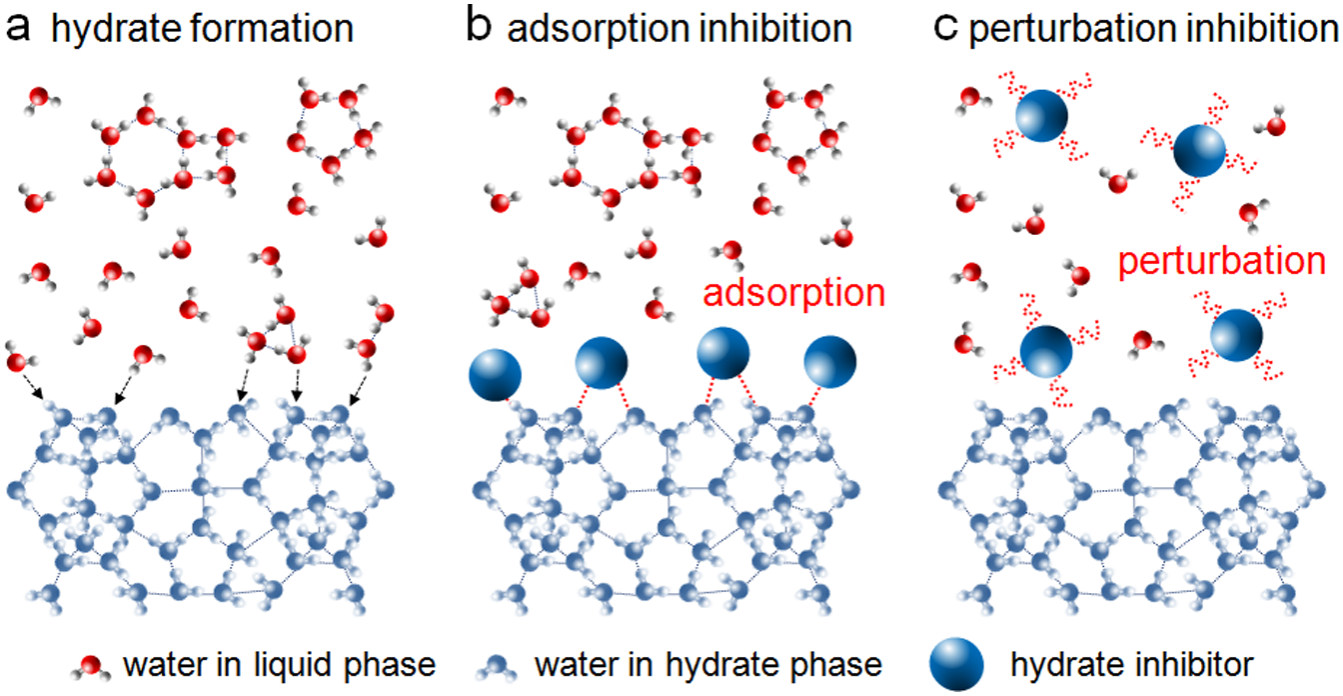
A schematic diagram demonstrating hydrate formation and the inhibition processes. Water molecules in liquid phase are connected through a hydrogen bond network (dashed line). (a) In the system without inhibitor, liquid water molecules close to the hydrate surfaces (*e.g.* nuclei and bulk surfaces) or solid substrates (*e.g.* reactor walls, foreign impurities) participate in hydrate formation. (b) The adsorption inhibition hypothesis involves adsorption of the inhibitors on the hydrate surface or any nucleating sites, inhibiting hydrate formation. (c) The perturbation inhibition hypothesis involves perturbation of the organization of local water molecules, preventing hydrate formation.

**Table 1:** The physicochemical properties of hydrophobic amino acids

	glycine (gly)	alanine (ala)	valine (val)	leucine (leu)	isoleucine (ile)
Molecular structure	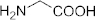	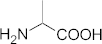	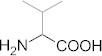	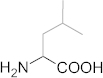	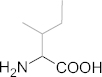
Side chain	-H	-CH_3_	-CH(CH_3_)_2_	-CH_2_CH(CH_3_)	-CH(CH_3_)C_2_H_5_
pK_a1_ (-COOH) at 273.15 K	2.41	2.39	2.33	2.39	2.38
Degree of ionization (-COOH) at pH 3.29	88.3%	88.7%	90.2%	88.9%	89.0%
pK_a2_ (-NH_2_) at 273.15 K	10.32	10.43	10.34	10.33	10.41
Degree of ionization (-NH_2_) at pH 3.29	100.0%	100.0%	100.0%	100.0%	100.0%
Hydrophobicity	−0.4	1.8	4.2	3.8	4.5

Acid dissociation constants at 273.15 K were calculated from the values listed in Table S1 using the van't Hoff equation. Hydrophobicity values were taken from the literature^60^.
