# Supplementary material for: Hydrophobic amino acids as a new class of kinetic inhibitors for gas hydrate formation
Source: Sci Rep. 2013 Aug 13;3:2428. doi: 10.1038/srep02428 (PMC3741619; doi:10.1038/srep02428)
Supplement: Supplementary Information [file srep02428-s1.doc]

**Supplementary Information for:**

**Hydrophobic amino acids as a new class of kinetic inhibitors for gas hydrate formation**

Jeong-Hoon Sa,^1^ Gye-Hoon Kwak,^1^ Bo Ram Lee,^2^ Da-Hye Park,^3^ Kunwoo Han,^4^ and Kun-Hong Lee^1*^

^1^*Department of Chemical Engineering, Pohang University of Science & Technology,*

*San 31, Hyoja-Dong, Nam-Gu, Pohang-Si, Gyeongbuk 790-784, Korea*

*^2^Center for Hydrate Research, Chemical and Biological Engineering Department, Colorado School of Mines, Golden, CO 80401, USA*

*^3^Energy Laboratory, Samsung Advanced Institute of Technology, San 14, Nongseo-Dong, Giheung-Gu, Yongin-Si, Gyeonggi-Do 446-712, Korea*

^4^*CO_2_ Project Team, Research Institute of Industrial Science & Technology,*

*San 32, Hyoja-Dong, Nam-Gu, Pohang-Si, Gyeongbuk 790-600, Korea*

*Corresponding author. Tel: 82-54-279-2271; Fax: 82-54-279-8298; e-mail: [ce20047@postech.ac.kr](mailto:ce20047@postech.ac.kr)

**1. Zwitterion formation of amino acids**

Four equilibrium reactions were involved in zwitterion formation of amino acids as shown below. Under neutral conditions, most of the carboxylic acid groups and amine groups were ionized into carboxylate ions (–COO^-^) and ammonium ions (–NH^3+^), respectively, as shown in Fig. S1. However, in high pressure CO_2_, the acidic environment was seen to influence their ionization behavior. Following theoretical calculations according to methods described in previous work,^1^ the pH during CO_2_ hydrate nucleation and growth in this work was estimated to be approximately 3.29, while the temperature and pressure were assumed to be 273.15 K and 32.8 bar, respectively, as an extreme case. At pH 3.29, almost 100% of the amine groups were expected to be ionized into ammonium ions while around 90% of the carboxylic acid groups were estimated to be ionized into carboxylate ions (Table 1).

$$NH_{2}RCOOH\leftrightarrow NH_{3}^{+}RCOO^{-}$$

$$K_{0}=\frac{a(HA^{\pm})}{a(HA)}$$

$$NH_{3}^{+}RCOOH\leftrightarrow H^{+}+NH_{3}^{+}RCOO^{-}$$

$$K_{a1}=\frac{a(HA^{\pm})a(H^{+})}{a(H_{2}A^{+})}$$

$$NH_{3}^{+}RCOO^{-}\leftrightarrow H^{+}+NH_{2}RCOO^{-}$$

$$K_{a2}=\frac{a(A^{-})a(H^{+})}{a(HA^{\pm})}$$

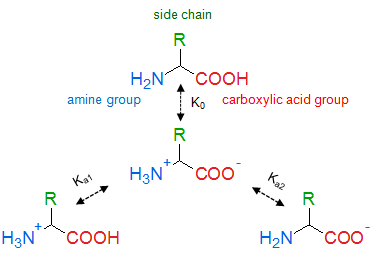


**Figure S1. Zwitterion formation and acid–base equilibria of amino acids in water.**

**2.Acid dissociation constant calculations**

The values of pK_a1_ and pK_a2_ for the hydrophobic amino acids at 298.15 K were first obtained from the literature, along with the heat of protonation values for the terminal carboxylic acids and amine groups.

**Table S1. Acid dissociation constants and heat of protonation values for hydrophobic amino acids at 298.15 K**

|  | glycine  (gly) | alanine  (ala) | valine  (val) | leucine  (leu) | isoleucine  (ile) |
| --- | --- | --- | --- | --- | --- |
| pK_a1_ (-COOH)  at 298.15 K^a^ | 2.34 | 2.34 | 2.32 | 2.36 | 2.36 |
| heat of protonation (-COOH)  at 298.15 K (kJ/mol) | 4.44^b^ | 3.35^c^ | 0.33^c^ | 1.76^c^ | 1.26^c^ |
| pK_a2_ (-NH_2_)  at 298.15 K^a^ | 9.60 | 9.69 | 9.62 | 9.60 | 9.68 |
| heat of protonation (-NH_2_)  at 298.15 K (kJ/mol) | 44.66^b^ | 46.19^c^ | 44.94^c^ | 45.61^c^ | 45.31^c^ |

^a^ Numerical values were taken from the literature^2^

^b^ Numerical values were taken from the literature^3^

^c^ Numerical values were taken from the literature^4^

From the thermodynamic data shown in Table S1, the values of pK_a1_ and pK_a2_ for the hydrophobic amino acids at 273.15 K were calculated using the van’t Hoff equation. In this step, it was assumed that the heat of protonation values were almost constant in the range of 273.15 to 298.15 K. The results of the calculations are summarized in Table 1.

$$K(T)=K(T_{0})\exp\left[-\frac{\Delta H}{R}\left(\frac{1}{T}-\frac{1}{T_{0}}\right)\right]$$

**3. Previous reports on perturbation of the local water structure around solutes**

A list of previous investigations into perturbation of the local water structure around solid solutes is given in Table S2.

**Table S2. Previous reports on perturbation of local water structure around solid solutes**

| Solute | Investigation tool | Research group | Reference |
| --- | --- | --- | --- |
| Hydrophobic amino acids | Infrared spectroscopy | The Scripps Research Institute | Hecht *et al.* Correlating hydration shell structure with amino acid hydrophobicity. *J. Am. Chem. Soc.* **115**, 3336-3337 (1993). |
| N-acetylamide with amino acid side chains | Neutron scattering | University of California, Berkeley | Pertsemlidis *et al.* Direct evidence for modified solvent structure within the hydration shell of a hydrophobic amino acid. *Proc. Natl. Acad. Sci. USA* **93**, 10769-10774 (1996). |
| Nonpolar solutes (CH_4_, -CH_3_) & polar or ionic solutes (Cs^+^, -OH) | Monte Carlo simulation | University of Pennsylvania | Sharp *et al.* Hydrophobic effect, water structure, and heat capacity changes. *J. Phys. Chem. B* **101**, 4343-4348 (1997). |
| Amino acids | Raman spectroscopy | Toyama University | Ide *et al.* Effect of hydrophobicity of amino acids on the structure of water. *J. Phys. Chem. B* **101**, 7022-7026 (1997). |
| N-methylpyrrolidone | Molecular dynamics simulation | University of Reading | Carver *et al.* Molecular dynamics calculations of N-methylpyrrolidone in liquid water. *Phys. Chem. Chem. Phys.* **1**, 1807-1816 (1999). |
| Quaternary ammonium zwitterions | Molecular dynamics simulation | University of Warwick | Storr *et al.* Kinetic inhibitor of hydrate crystallization. *J. Am. Chem. Soc.* **126**, 1569-1576 (2004). |
| Polyethylene oxide, polyvinyl pyrrolidone, polyvinyl caprolactam and methylvinylacetamide | Molecular dynamics simulation | Massachusetts Institute of Technology | Anderson *et al.* Properties of inhibitors of methane hydrate formation via Molecular Dynamics simulations. *J. Am. Chem. Soc.* **127**, 17852-17862 (2005). |
| Polyvinyl pyrrolidone and polydimethylamino-ethylmethacrylate | Molecular dynamics simulation | University of Warwick | Moon *et al.* Nucleation and control of clathrate hydrates: insights from simulation. *Faraday Discuss.* **136**, 367-382 (2007). |

**4. Nucleation kinetics measured by the isothermal method**

When 1.0 mol% glycine was injected into the system, the average induction time was extremely long compared to that for other measurements (Fig. S2a). However, there was no clear trend evident with variation in glycine concentration, and the calculated standard deviations of induction time were too large, with values ranging from 1 min to 55 h. Even for the measurements of the system with 0.5 wt% PVP, it was almost impossible to assess any delay in hydrate nucleation. This stochastic nature was also identified in the measurements involving the different hydrophobic amino acids, as shown in Fig. S2b. In addition, induction time measurements at lower subcooling (at higher temperatures) showed more scattered results (data not shown).

**
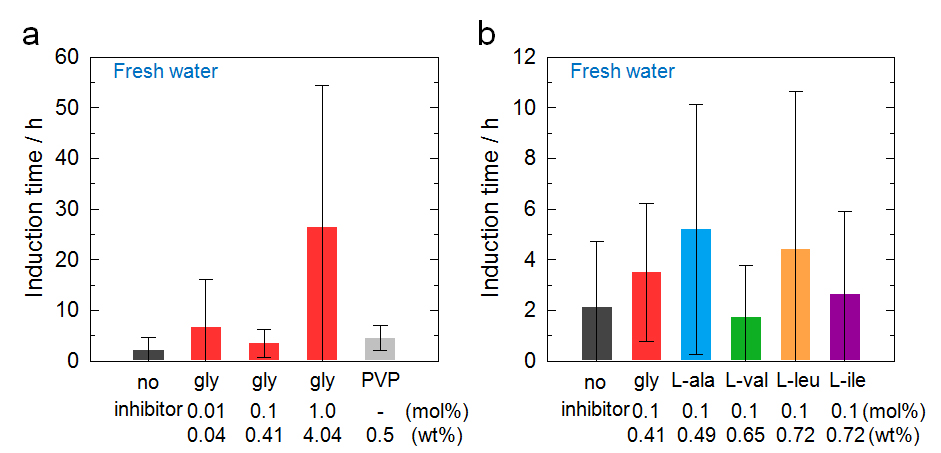
**

**Figure S2.** **Heterogeneous nucleation kinetics of the CO_2_ hydrates in the presence of hydrophobic amino acids.** Induction times for CO_2_ hydrate nucleation with (a) glycine or PVP in fresh water, and (b) hydrophobic amino acids in fresh water. The values represent the average ± standard deviation.

**5. Growth kinetics measured by the isothermal method**

Growth kinetics data for 10 h of hydrate formation are shown in Figure S3. While most of the systems demonstrated negligible increases in gas uptake from 2 to 10 h, some exhibited a more pronounced increase.


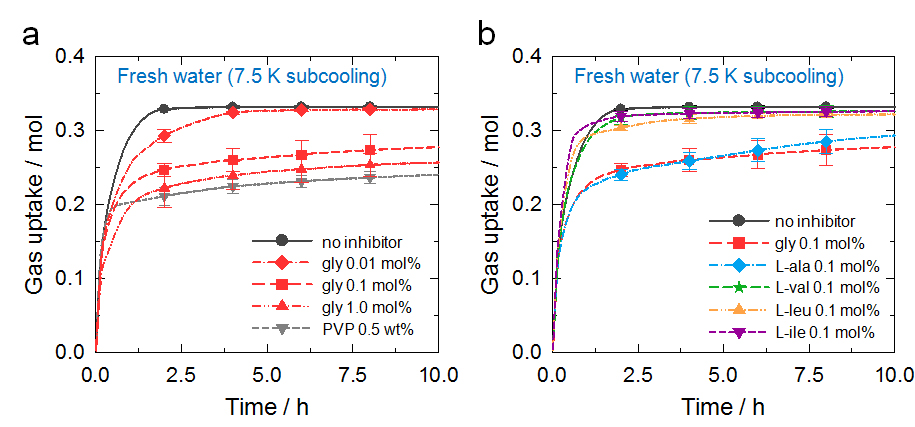


**Figure S3. Growth kinetics of the CO_2_ hydrates in the presence of hydrophobic amino acids.** Gas uptake rates during CO_2_ hydrate growth from 0 to 10 h (a) with glycine or PVP and (b) with hydrophobic amino acids. Measurement of hydrate growth was initiated just after the onset of hydrate nucleation. Average values of gas uptake were calculated from several repeating measurements taken every 10 s. The symbols and their error bars indicate the average and standard deviations of gas uptake at each time point, respectively.

**6. Synchrotron PXRD**

The structure of CO_2_ hydrate was confirmed to be structure I from 25 diffraction peaks at 1 bar and 80.0 K. Using the Chekcell program, the observed and calculated values of 2θ for CO_2_ hydrates were obtained and are summarized in Table S2, from which the error can be estimated. Although error estimations for all samples were carried out, the result for pure CO_2_ hydrate is shown as a representative value.

**Table S3. The observed and calculated 2θ values for CO_2_ hydrate from PXRD**

| peak  number | *h k l* | 2θ_observed_ | 2θ_calculated_ | 2θ_observed-calculated_ |
| --- | --- | --- | --- | --- |
| 1 | 2 0 0 | 14.960 | 14.972 | -0.012 |
| 2 | 2 1 1 | 18.365 | 18.364 | 0.001 |
| 3 | 2 2 0 | 21.235 | 21.235 | 0.000 |
| 4 | 3 1 0 | 23.765 | 23.776 | -0.011 |
| 5 | 2 2 2 | 26.075 | 26.084 | -0.009 |
| 6 | 3 2 0 | 27.170 | 27.169 | 0.001 |
| 7 | 3 2 1 | 28.215 | 28.215 | 0.000 |
| 8 | 4 0 0 | 30.210 | 30.208 | 0.002 |
| 9 | 4 1 0 | 31.140 | 31.161 | -0.021 |
| 10 | 4 1 1 | 32.070 | 32.089 | -0.019 |
| 11 | 4 2 0 | 33.875 | 33.875 | 0.000 |
| 12 | 4 2 1 | 34.745 | 34.738 | 0.007 |
| 13 | 3 3 2 | 35.590 | 35.583 | 0.007 |
| 14 | 4 2 2 | 37.230 | 37.222 | 0.008 |
| 15 | 4 3 0 | 38.030 | 38.018 | 0.012 |
| 16 | 5 1 0 | 38.810 | 38.801 | 0.009 |
| 17 | 5 2 0 | 41.060 | 41.074 | -0.014 |
| 18 | 5 2 1 | 41.805 | 41.808 | -0.003 |
| 19 | 5 3 0 | 44.660 | 44.649 | 0.011 |
| 20 | 5 3 1 | 45.330 | 45.337 | -0.007 |
| 21 | 6 0 0 | 46.035 | 45.016 | 0.019 |
| 22 | 6 1 0 | 46.690 | 46.688 | 0.002 |
| 23 | 6 1 1 | 47.345 | 47.353 | -0.008 |
| 24 | 6 2 0 | 48.645 | 48.662 | 0.017 |
| 25 | 6 2 1 | 49.295 | 49.306 | -0.011 |

**7. Lattice parameters**

Calculated lattice parameter values for each sample were calculated from the 25 diffraction peaks using the Chekcell program. The addition of neither hydrophobic amino acids nor PVP altered the crystal structure of the CO_2_ hydrate.

**Table S4. Calculated lattice parameters for CO_2_** hydrate with hydrophobic amino acids or PVP.

| system | lattice parameter (Å) |
| --- | --- |
| no inhibitor | 11.8776 ± 0.0076 |
| gly 0.1 mol% | 11.8814 ± 0.0053 |
| gly 1.0 mol% | 11.8807 ± 0.0036 |
| PVP 0.5 wt% | 11.8867 ± 0.0081 |
| L-ala 0.1 mol% | 11.8831 ± 0.0076 |
| L-val 0.1 mol% | 11.8830 ± 0.0086 |
| L-leu 0.1 mol% | 11.8854 ± 0.0106 |

**8. Materials**

The deionized water used in this work was distilled in the laboratory. CO_2_ with ultra-high purity (99.999%) was supplied by Deokyang Energen Corporation, Korea. All chemicals were of reagent grade (≥98%) and were used without further purification. Glycine, L-alanine, L-valine, L-leucine, L-isoleucine, and PVP were obtained from Sigma-Aldrich, USA. The PVP had an average molecular weight of approximately 29,000.

**9. Experimental apparatus**

The experimental system used in this work was similar to that reported in our previous work.^1^ Macroscopic measurements and sample preparation for microscopic analysis were carried out in a high pressure cell made of SUS 316 with a volume of 250 cm^3^ and a maximum operating pressure of 80 bar. The cell was immersed in an ethanol bath and the maximum cooling rate of the system was 0.25 ± 0.05 K/min. The solution in the cell was agitated by an impeller coupled to a magnetic drive. The temperature and pressure in the cell were recorded every 10 s with a K-type thermocouple and a WIKA A-10 pressure transmitter within a maximum error of ± 0.1 K and ± 0.5%, respectively.


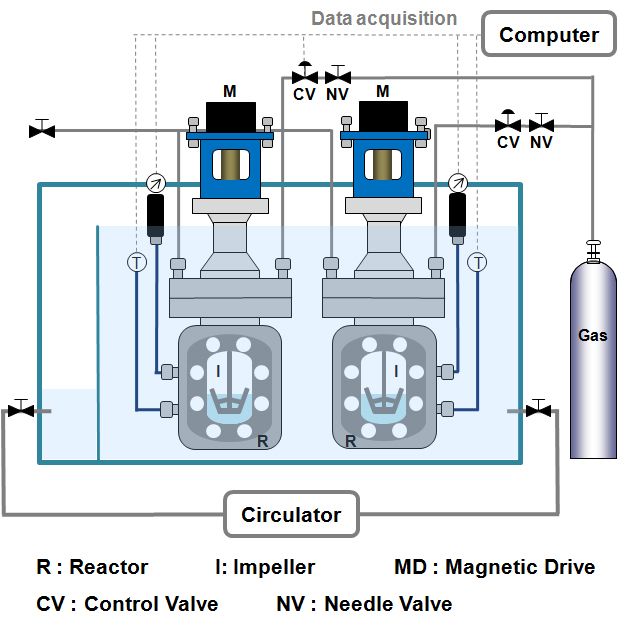


**Figure S4. Schematic diagram of the experimental system.**

**10. Experimental procedures for macroscopic measurements**

Temperature and pressure traces during macroscopic measurements are shown below.


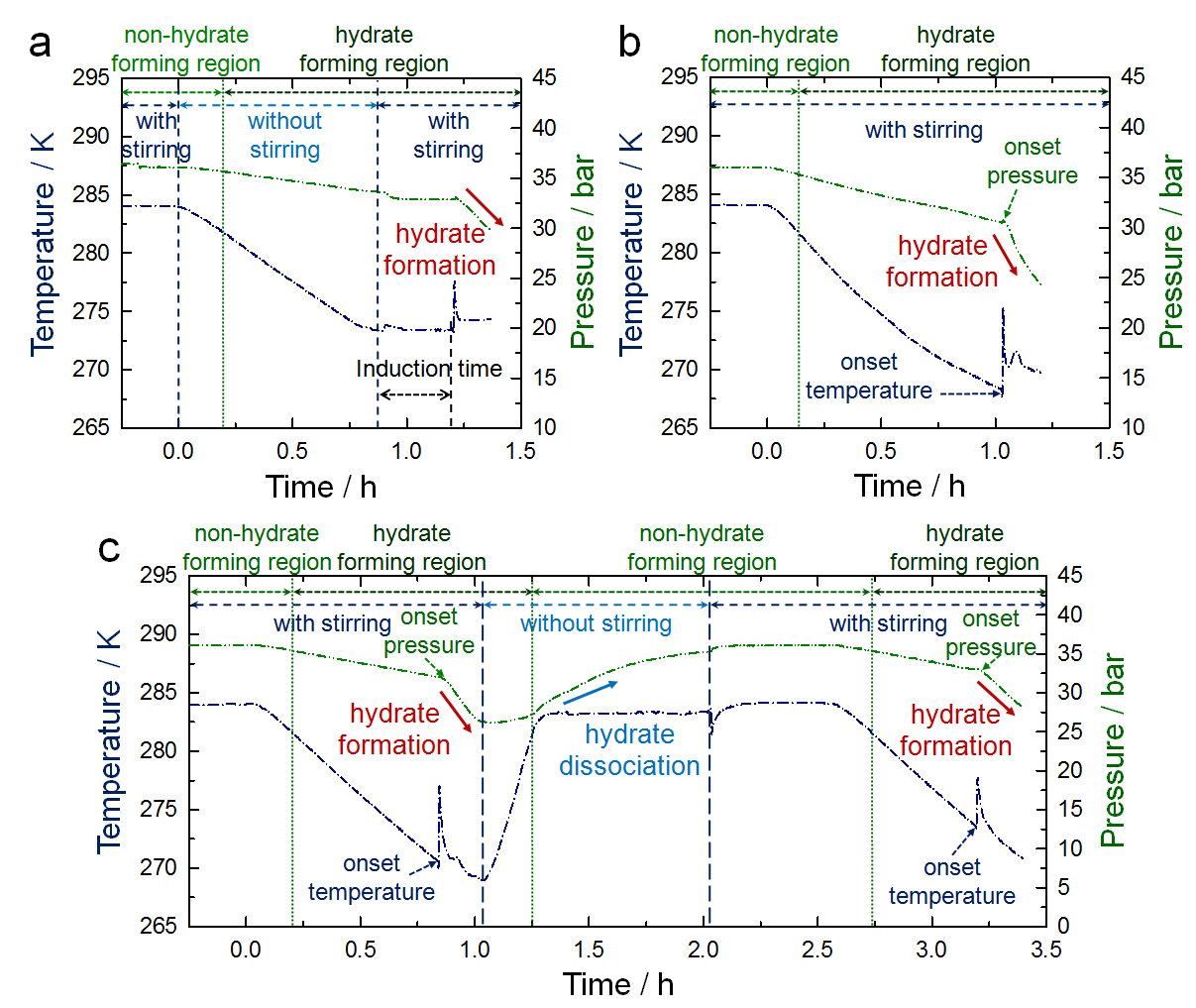


**Figure S5. Experimental procedures for macroscopic measurements.** Temperature (blue) and pressure (green) change during experiments using (a) isothermal, (b) constant cooling, and (c) constant cooling with superheated hydrate methods.

**11. Experimental procedures for synchrotron PXRD at 9B high-resolution powder diffraction beamline in Pohang Accelerator Laboratory**

The powder sample was loaded on a sample holder, and then vacuumized. After beam injection at 80 K, a step scan was conducted.


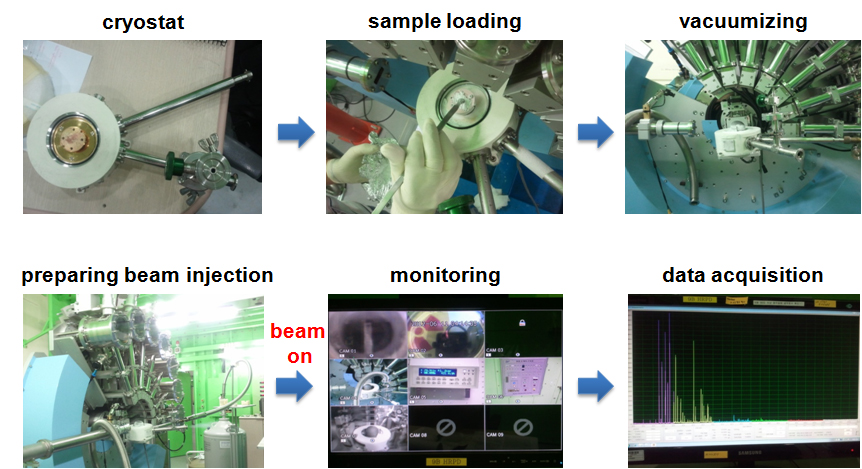


**Figure S6. Experimental procedures for synchrotron PXRD analysis.**

**References**

1. Sa, J.-H., Lee, B. R., Park, D.-H., Han, K., Chun, H. D. & Lee, K.-H. Amino acids as natural inhibitors for hydrate formation in CO_2_ sequestration. *Environ. Sci. Technol.* **45**, 5885-5891 (2011).
2. Bruice, P. Y. *Organic Chemistry* (Pearson Education, Upper Saddle River, NJ, 2007).
3. Kiss, T., Sovago, I. & Gergely, A. Critical Survey of stability constants of complexes of glycine. *Pure Appl. Chem.* **63**, 597-638 (1991).
4. Smith, P. K., Taylor, A. C. & Smith, E. R. B. Thermodynamic properties of solutions of amino acids and related substances. III. The ionization of aliphatic amino acids in aqueous solution from one to fifty degrees. *J. Biol. Chem.* **122**, 109-123 (1937).
